# Performance of plasma biomarkers for diagnosis and prediction of dementia in a Brazilian cohort

**DOI:** 10.1002/alz70856_101173

**Published:** 2025-12-25

**Authors:** Luis E. Santos, Paulo E. Mattos, Thaís Lopes Pinheiro, Ananssa Silva, Claudia Drummond, Felipe K. Sudo, Fernanda G. Q. Barros‐Aragão, Bart Vanderborght, Carlos O Brandão, Sergio T. Ferreira, Fernanda Tovar‐Moll, Fernanda Guarino De Felice, IDOR Memory Clinic Initiative

**Affiliations:** ^1^ D'Or Institute for Research and Education, Rio de Janeiro, RJ, Brazil; ^2^ D'Or Institute for Research and Education (IDOR), Rio de Janeiro, RJ, Brazil; ^3^ Federal University of Rio de Janeiro (UFRJ), Rio de Janeiro, RJ, Brazil; ^4^ Neurolife, Rio de Janeiro, RJ, Brazil; ^5^ Queen's University, Kingston, ON, Canada; ^6^ Federal University of Rio de Janeiro, Rio de Janeiro, RJ, Brazil

## Abstract

**Background:**

Dementia is a growing concern throughout the developing world and is severely underdiagnosed among the Brazilian population. Despite remarkable progress in the biomarker field in recent years, local testing and validation of plasma biomarkers of AD and dementia is still lacking in Brazil and Latin America.

**Methods:**

In this longitudinal cohort study of 145 participants, the diagnostic performance of plasma biomarkers was assessed based on clinical diagnosis and CSF biomarker positivity. Follow‐up data of up to 4.7 years were used to determine biomarker performance in predicting diagnostic conversions. The study was conducted at the Memory Clinic at the D’Or Institute for Research and Education (IDOR) in Rio de Janeiro. Participants were volunteers referred to the service. All were native Brazilians, had Portuguese as their first language and 60+ years of age. They were clinically diagnosed and underwent psychiatric and laboratory assessments. Diagnoses outside the scope of the study were excluded. CSF biomarker data was available for 36% of the sample. Participants were categorized as cognitively unimpaired (CU; *n* = 49) or cognitively impaired (CI; *n* = 96), with CI participants further classified as aMCI, Alzheimer's disease (AD), Lewy body dementia, or vascular dementia. Plasma Tau, Aβ_40_, Aβ_42_, NfL, GFAP, pTau231, pTau181 and pTau217 were measured at initial and follow‐up visits using a SIMOA HD‐X instrument. To assess the diagnostic performance of each biomarker, receiver operating characteristic (ROC) curve analysis was performed.

**Results:**

The pTau217/Aβ_42_ ratio was the top‐performing biomarker for discrimination between CSF‐biomarker‐negative and CSF‐biomarker‐positive subjects (AUC=0.98, 95% CI: [0.94 – 1.00]), followed by pTau217 alone (AUC=0.94, 95% CI: [0.88 – 1.00]. When discriminating participants based on their cognitive status (CU x CI), pTau217 and pTau217/Aβ_42_ ratio reached identical AUCs of 0.82 (95% CI: [0.75 – 0.89]). Similar results were seen when discriminating CU from all‐cause dementia, with pTau217 and pTau217/Aβ_42_ ratio tied at the highest AUCs (AUC=0.87, 95% CI: [0.80 – 0.94]).

**Conclusions:**

In this first biomarker profiling of a Brazilian dementia cohort, plasma pTau217 confirmed its potential as a clinically useful diagnostic tool. This study comprises an initial step towards local validation and adoption of dementia biomarkers in Brazil and Latin America.

